# Book Reviews

**Published:** 1800-07

**Authors:** 


### CRITICAL RETROSPECT

OF

### MEDICAL AND PHYSICAL LITERATURE.

### [FOREIGN AND DOMESTIC.]

*Tranfactions of a Society for the Improvement of Medical and Chirurgical Knowledge*. Illuftrated with copper-plates. Vol. II. 8vo. pp. 380. *London*, Johnfon. 1800.

THE defign of this work is fimilar to that of Duncan's Annals of Medicine, or Dr. Simmons's Medical Facts. It appears to be the intention of the editors to admit original papers only; and while they are fupplied by fuch names as ornament the prefent volume, there can be no doubt that the work will maintain its popularity.

This volume contains twenty-nine papers on fome of the moft interefting fubjects of medicine and furgery, from which we felect the following extracts.

*Account of a Cafe in which Death was brought on by a Hæmorrhage from the Liver.* By G. BLANE, M. D. F. R. S. &c. *Read May 6,* 1794.

" A BOY, aged eight years, of a delicate conftitution, fmall ftature, and pale complexion, whofe family on the father's fide had been extremely fubject to phthifis pulmonalis, but he himfelf having no fymptoms of fcrofula or rickets, or any external blemifh or deformity, except that his belly had always been too large, and he had been fubject to complaints of the ftomach and bowels through life, began to complain on the 12th of March, 1794, of languor, lofs of appetite, and flight pains, which he referred to his breaft and ftomach. Thefe fymptoms continued the fame next day, but became more fevere on the 14th, and remained fo the three following days, on the laft of which he became feverifh, and there was an eruption of red fpots on the neck and breaft, which lafted only a few hours. On the 18th and 19th, the fymptoms were milder, and he was thought to be recovering; but in the night between the 19th and 20th, he was feized with a fevere pain, which he referred to the left hypochondrium. This continued between five and fix

 hours, hours, and he then became fuddenly faint, and lofing all fenfe and motion was thought to be in a fwoon, but from this he never recovered.

" This is the account which I received from the family and the medical gentleman who attended, for I did not fee him during his illnefs; and not having been fent for till he was in extremities, he had died before I arrived.

" Leave was obtained to examine the body, and the following appearances were obferved. Upon laying open the abdomen, a large quantity of coagulated blood was difcovered, covering the whole left fide of the inteftines. In exploring the fource of this, feveral fiffures were obferved in the left lobe of the liver, which were about two-thirds of an inch in length, whence the blood had undoubtedly flowed, for they were found to lead to a cavity in the fubftance of the lobe of about the fize of a pigeon's egg, and full of blood. The peritonæum, on the furface of that part of the liver which was near one of the fiffures, was raifed from the liver like a blifter, full of coagulated blood. Thefe appearances were on the lower furface of the lobe, but on the upper furface of the fame lobe there was one fiffure, round which was a fimilar collection of blood between the peritonæum and the fubftance of the liver, and it led to a fmall bloody cavity near the furface of the liver.

" There was no external livor, or other mark of injury either on the adjacent *parietes*, or on the fpleen or inteftines.

" It feems moft probable therefore, that the rupture and confequent hæmorrhage were owing to the weak ftructure of the liver, which correfponded with the general weaknefs of this young perfon's frame.

" It would appear that it is this weaknefs, proceeding from a too great tenuity of the coats of the veffels, that chiefly conftitutes the fcrofulous habit, of which the principal fymptoms feem referrible to a rupture of the fmaller feries of veffels either circulatory or lymphatic, and the confequent effufion either of red blood or colourlefs fluids, occafioning fometimes hæmorrhages, but more frequently interftitial or glandular depofits. The fine fkin and filky hair obfervable in fuch habits are farther in proof of this.

" As far as my reading goes, I have not met with any morbid affection of the liver fimilar to that above defcribed, or have I ever feen or heard of fuch a cafe, which has made me think that it might deferve a place in fome regifter of facts. It has indeed been alleged, that there is not much utility in recording extraordinary and anomalous cafes, as they are feldom the objects of practice. But if there is any juftice in this remark, it applies rather to unufual combinations of fymptoms, than to morbid appearances detected by diffection; for as the number of bodies that are infpected is comparatively fmall, there is reafon to prefume, that, in the numberlefs others which are not examined, there may be many among them fimilarly affected."

The

The third paper contains an elaborate

*Account of the Croup, as it appeared in the Town and Neighbourhood of Cheſham, in Buckinghamſhire, in the Years* 1793 *and* 1794. By HENRY RUMSEY, Surgeon at Cheſham.

IN this account the author ſees reaſon for admitting the exiſtence of *ſpaſmodic* croup as well as inflammatory; and he ſays,

"It appears to me that the croup is an inflammation of its own kind. If it conſiſted in common inflammation, we might expect to find the ſame appearances (that is, the ſame kind of concretion on the ſurface of the trachea) every day, as its mucous membrane is ſo frequently the ſubject of inflammation attended with an increaſed ſecretion. The matter, however, of which this ſubſtance is formed, poſſeſſes different properties from thoſe of the mucous which is thrown out upon the membrane of the noſe, or of the trachea in common catarrhal affections.

"I think it probable, that the film which we find in the croup is not formed by a ſecretion from the mucous glands, but is an exſudation from the exhalant arteries. Upon this principle we can more eaſily account for ſuch film not being found in common catarrhal affections, in which the mucous glands are perhaps more the ſeat of the diſeaſe. It is, therefore, analogous to the inflammatory exſudation in the inflammation of other internal membranes firſt deſcribed by the late Dr. Hunter.

"The croup has been ſometimes thought infectious, but I have not been able to form a decided opinion upon this point. Some circumſtances render it probable, as two and ſometimes three children in the ſame family have been ſeized with it. But on the other hand, I have at different times ſeen two or three in a family eſcape, while one or two of the others have died of it, without any pains being taken to keep thoſe who were in health from the ſick. When a diſeaſe is epidemic, it is ſometimes difficult to determine whether it be communicated by infection, or whether ſeveral people have the diſeaſe in conſequence of their being expoſed to the ſame exciting cauſe. It is rather remarkable, that although there were between twenty and thirty children in our workhouſe, only one had the diſeaſe."

In the treatment the author thinks that calomel, in alterative doſes, as recommended by Dr. Ruſh, proved a uſeful remedy.

*An Account of two Cases, ſhewing the Existence of the Small-Pox and the Measles in the same Person at the same Time.* By P. RUSSEL, M. D. F. R. S.

"THE Meaſles and the Small-pox were epidemical at Aleppo, in the year 1765. They both made their appearance about the ſame time in December of the preceding year, and gradually increaſed through January, February, and part of March. The Meaſles diſappeared in April, while the Small-Pox lingered till the ſummer ſolſtice.

"Till the month of March, both diſeaſes were, in general, of a favourable kind. The eruption in the Meaſles commonly happened pened on the evening of the third, or morning of the fourth day. The eruptions were firſt viſible on the face; and began to fade every where on the ſixth or ſeventh day, but ſeldom diſappeared entirely ſooner than the tenth. They were rather more prominent than thoſe which I had obſerved in Europe, and the branny ſkurf left on the ſkin was conſiderably leſs: in other reſpects there was no material variation from Sydenham's Accurate Deſcription of the Meaſles, epidemic at London in the Year 1670.

"After the beginning of March, the diſeaſe became more formidable. The eyes were more inflamed, the coryza, the cough, and the febrile ſymptoms were in a greater degree than in the former months. The eruption appeared irregularly from the ſecond to the ſeventh day of the fever. The eruptions often appeared on the breaſt before they were ſeen on the face; they were of a fainter colour, and the branny ſkurf was, in general, leſs. A vomiting and diarrhœa were common ſymptoms from the beginning; the latter continued throughout the diſeaſe; the former uſually ceaſed as ſoon as the eruption was complete: but very young ſubjects continued to be harraſſed by retchings, excited by the irritation of ſneezing or coughing.—About the height of the diſeaſe, the ſick were ſubject to ſhort exacerbations of the fever, accompanied with difficulty of breathing, and ſtitches in the cheſt, which recurred ſeveral times in the twenty-four hours. On the whole, the Meaſles, after the month of February, approached nearer to the anomalous ſpecies deſcribed by Sydenham under the year 1674.

"From the end of February, the Small-pox, which hitherto had been of a mild diſtinct kind, became irregular and much more fatal. The puſtules were confluent on the face; on other parts diſtinct, but generally flattiſh, and indented at the top. In many caſes they ſuppurated imperfectly; in others, they remained dry or huſky: and ſome inſtances occurred of their turning black about the ſeventh day. A diarrhœa and teneſmus frequently attended from the beginning to the end.

"Though a large proportion of the infected ſtill recovered, they continued long in a ſickly ſtate. Children were not only reduced by the diarrhœa, but were often attacked with the hooping-cough, or with erratic fevers, both being at that time epidemic in the city. In the more malignant kind of Small-pox, many died between the ſeventh and ninth day; ſome ſtruggled to the eleventh; very few recovered.

"When the Small-pox and the Meaſles prevailed in the ſame ſeaſon, many of the children ſuffered both diſeaſes in ſucceſſion, as uſual. The Meaſles were rarely obſerved to ſucceed the Small-pox in leſs than twenty days, reckoning from the eruption. The Small-pox commonly ſucceeded the Meaſles ſomewhat earlier in the third week; but ſeveral caſes were met with, in which the puſtules of the Small-pox were diſcovered on the face, before the total diſappearance of the Meaſles on the limbs; that is, on the eleventh or twelfth day.

"It

"It has already been remarked, that few of the infected in either difeafe died before the month of March. But from that time the mortality in the Small-Pox was confiderable; as thofe who had juft before fuffered from the Meafles, and were reduced by the diarrhœa to a ftate unfavourable for the reception of a fatiguing diftemper, generally perifhed; unlefs the fupervening Small-pox was of a mild kind. The danger appeared to be rather lefs where a bad kind of Meafles fucceeded the Small-pox.

"The reciprocal influence of the two difeafes in the fame fubject was carefully attended to in above three hundred cafes; and fo little did the quality of the firft difeafe feem to influence that of the fecond, that a mild, diftinct Small-pox was often obferved to follow the worft kind of Meafles, and vice verfa.

"In the month of March, an inftance occurred where both difeafes were conjoined in the fame patient. The fubject was a female child two years old, of a pale, delicate complexion. The rednefs of the eyes, the coryza, and the cough which accompanied the fever, led me to expect the Meafles. On the fourth day, the eruptions of the Meafles were vifible on the face, the neck, and the back; but at the fame time a few eruptions of a different kind were interfperfed on the face and neck, which, if they had been the fole eruption, I fhould without hefitation have declared to be the Small-pox. The progrefs of the puftules on the fifth proved them to be variolous. Both eruptions were of a favourable kind, and diftinctly purfued their regular courfe. On the eighth day, the Meafles were fading faft, while the variolous puftules on the face were near their height. The puftules were not numerous, were very diftinct, and ripened perfectly. The cough continued to be a troublefome fymptom, efpecially in the fecond week. A diarrhœa fupervened about the fourteenth day, and contributed to render the child's recovery very flow.

"In the month of April I met with a fimilar cafe. A healthy boy, three years old, was attacked with the ufual fymptoms of the eruptive fevers, at that time epidemical. The cough rather feemed to indicate the Meafles. On the third day, the eruptions of the Small-pox and Meafles made their appearance together. The variolous puftules were of the fmall, round kind, and came to perfect maturity; but were more numerous than in the former cafe. The Meafles were of a fainter colour, and left behind them ftill lefs of the branny fkurf, agreeing in both circumftances with the difeafe then prevalent.

"On the fixth day of the fever, a diarrhœa and tenefmus were joined to the haraffing cough, and reduced the patient fo low in the fecond week, that his life was defpaired of. The flux, however, leaving him towards the end of the month, he recovered."

---

*An Addrefs to the Public on the Advantages of Vaccine Inoculation: with the Objections to it refuted.* By HENRY JENNER, Surgeon, F. L. S. &c. 4to. pp. 20; London, Cadell and Davies.

THE author commences his *addrefs* by ftating, "in the way of comparifon, comparifon, the peculiar differences which mark the Small-pox and Cow-pox;" and he affirms that the ftatement is " in every particular, confirmed by very extenfive experience."

After the comparifon of the advantages which are to be derived from the fubftitution of the Vaccine Difeafe for the Small-pox, the author obferves, " It is perfectly confiftent with my prefent defign, briefly to notice the moft popular objections which have been urged againft the introduction of Cow-pox.

" It has been called a beftial humour, and by a fallacious affociation of ideas, it is fuppofed to introduce an *unnatural* difeafe into the conftitution.

" If this very weak and futile objection were worthy of reply, we might obferve, that the cow is of all others the moft healthy and the moft cleanly of our domeftic animals, and might alfo remark, that no females are fo healthy as our dairy maids, whofe morning and evening hours are fpent amongft the cows; and we fhould not forget that eminent phyficians recommend invalids to avail themfelves of the falubrious effects of the breath of the heifer. How void of foundation then muft be the objection to the infertion of an atom of matter taken from the teat of the cow, once in the life only, when every perfon is in the daily habit of introducing into his ftomach various parts of the fame animal. The human ftomach revolts not at beef, butter, cheefe, and cream; yet every one, acquainted with the animal economy, muft know, that thefe aliments are quickly mingled with the conftitution.

" Another grand objection (which indeed is the only one that ftrikes at the *foundation* of our theory) is, that perfons are liable to be affected with fmall-pox after having been inoculated with the Cow-pox.

" A very extenfive practice, and an equally extenfive communication of the experience of medical friends of the firft reputation, would almoft warrant a fhort, abrupt anfwer to this queftion; an anfwer conveyed in terms unaccommodated to the feelings of the prefent age. After this objection, which has been refuted as often as it has been urged, a laconic reply, conveyed in no polite language, would by no means be improper, as it would harmonize with the general mode of fuch objections; and by all laws, the anfwer ought to be of the colour of the queftion; but what is not owing to cavilling individuals, is a juft debt due to a candid and judicious public.

" Every cafe that has been brought forward to undermine the theory we defend, we can prove to a demonftration was not one of the *genuine* kind. There are *three* difeafes which have indifcriminately been termed Cow-pox, only *one* of which is the real preventive of fmall-pox. In the fpring feafon particularly, cows are frequently fent to market for fale: the farmer omits to milk them in the morning, previous to their fetting out, that their udders may appear full, and the animals on that account become more valuable. The frequent confequence is, that inflammation enfues, which terminates in eruptions on the teats and udder, and affects the the milker with a loathfome difeafe on the hands, arms, and fhoulders. The forehead fometimes does not efcape, from the circumftance of the fervant's leaning againft the udder in milking. This difeafe may affect the fame perfon feveral times, but it will *never* prove a preventive for Small-pox: a cafe of this kind occurs in the city of Briftol; a Mr. Jacobs, attorney at law, was extenfively affected *twice* with this difeafe (which, from his total ignorance of *real* Cow-pox, he has called by that name,) but it did not prevent his being afflicted with a fubfequent fevere Small-pox.

---

*Syfteme Methodique de Nomenclature, &c.*—A Methodical Syftem of Nomenclature and Claffification of the Mufcles of the Human Body, with Defcriptive Tables, fhewing their Old and New Names, Situations, Attachments, Direction, Compofition, Figure, Connexion, and their Ufes: To which is added, a Dictionary, containing the Synonyms of the Mufcles. By C. L. DUMAS, Profeffor of Anatomy and Phyfiology, &c. &c. *Montpelier.* 4to. 200 pp.

IT is almoft univerfally allowed that the anatomical terms which are at prefent in general ufe, are very imperfect, and by no means correfpond with the numerous and ufeful difcoveries in this fcience; an attempt to correct thefe defects will not now be confidered fo daring an innovation as it might have been formerly. Mons. Dumas, as profeffor of anatomy, was conftantly remarking the difficulty of teaching this fcience from the crowd of abfurd names given to various parts; and has undertaken in this work, the very arduous and ufeful tafk of rectifying them; and though he may not have fully anfwered our expectations, yet we think a tranflation of this work, with fome alterations that would occur on a careful perufal, would be a very acceptable prefent to the ftudents of anatomy of this country.

In the third chapter, the author confiders fome of the defects of anatomical language in general, and the mode of correcting it; and we tranflate the following as a fpecimen of his manner of treating the fubject.

" A flight examination of the moft common anatomical terms will be fufficient to fhew the incorrectnefs and impropriety of a great number of them. There are fome which are ftrictly arbitrary, and others which have no determined fignification, and which any anatomift may change at pleafure. Under this clafs may be ranged all thofe denominations drawn from numerical orders, &c. which exprefs neither fituation, form, figure, nor connexion; but only the number of parts, which does not define any thing *anatomically*. Of courfe, we reject the names of firft, fecond, and third phalanx, &c. terms given to bones which have no agreement or relative fize. *Vefalius's* terms of firft, fecond, third, fourth, fifth, fixth, &c. mufcles of the arm; *Columbus's* firft, fecond, and third mufcles of the *triceps*; *Winflow's* firft, fecond, and third *adductors* of the thigh; and and first and second *external radial*; and *similar* names given to muscles which differ in situation, attachment, and figure, must at once appear ridiculous.

"If anatomical terms have sometimes a vague and arbitrary signification, there are several that have no meaning at all. What sense can be annexed to *blind hole*, *ossa innominata*, *accessary muscle*, *sublime muscle*, *humble muscle*, *profound muscle*, *pudendal artery*, &c. &c. *Turks saddle*, *par vagum*, *recurrent nerve*, *nates*, *testes* & *vulva cerebri?* How is it possible to associate such ridiculous expressions with any true and clear ideas of anatomy?

"There are other names perhaps less absurd, although not more regular, which actually tell us what they do not mean—such are the names of *vena cava*, which is not the only vein that is hollow, and *ductus arteriosus* to distinguish a duct, which is actually ligamentous in the adult.—*Arbor vitæ*, to characterize the ramifications which the internal surface of the *cerebellum* presents when cut vertically, and in which nature has not exclusively placed the seat of life, &c.

"The frequent use of terms in an absolute sense, which are only relative, is an essential defect in anatomical language. The adjectives, *great*, *little*, *vast*, *thin*, *superior*, *inferior*, *short*, *long*, *right*, *left*, are examples of this impropriety. When we talk of the *great* and *little* wings of the sphenoid bone, the *great* and *little* condyles of the humerus, the *great* and *little* trochanters of the thigh, the *great* and *little* angle of the eye, and the *great* and *little* bones of the tarsus, it is impossible to comprehend them. The epithets of *great* bone, *grand* artery, *great* and *little* saphena, *great* and *little* sympathetic, *great* and *little* meseraic, can never be consonant to a proper nomenclature, and ought to be abolished, since great, little, long, short, &c. are not essential properties, but only qualities relative to a part, and can only serve in the description of one part."

It would occupy more room than we can possibly allow in our Journal to follow the author in his censures on all the absurd names that are in general use, as well as the idle vanity of denominating parts after the discoverers of them; who, the author justly observes, have no occasion for such auxiliaries to render their names immortal; not to mention the possibility of attributing a discovery to one person, when it actually belongs to another.

The author's plan is to divide the human body into forty-seven different regions, all of which have particular names, and under which all the muscles may be ranged in a natural order, and immediately found as indicated by their proper heads and classes; and he thinks this method will facilitate both the study and investigation of anatomy, by assembling together what ought to be united, and separating what ought to be divided, as putting each muscle in its proper place, and circumscribing its limits; and, although he says the same idea has been adopted by *Chaussier*, he thinks his own an improvement upon it.

To

To illuftrate his mode, the author has given us tables of the mufcles, arranged under diftinct columns according to their regions, with their old and new names, fituation, firft attachments, fixed points, direction, laft attachments, points of infertion, compofition and figure, connexion, and ufes. And at the end of the work he has added an ufeful dictionary of fynonyms of all the mufcles of the human body from the beft authors.

---

*Nordifhes Archiv. i. e. Northern Archives of Natural and Medical Science; or, Magazine for Medical and Natural Science of the North.* Edited by Prof. PFAFF, of Kiel, and Dr. SCHEEL, of Copenhagen. Firft Volume, firft Number, 1799. pp. 192, 8vo. Price 16 gr. or about 2s. 6d. Copenhagen, Brummer.

THE Editors of this new periodical publication propofe to collect all contributions relating to natural and medical fcience, by which both are, as well in general as in their fingle parts, illuftrated, enlarged, and corrected, and the practical application of them, for the prefervation of health, and cure of difeafes, really improved; but merely as far as this is owing to the induftry and genius of northern (Swedifh and Danifh, &c.) phyficians, furgeons, and naturalifts. We fhall be enabled, by this ufeful publication, to get acquainted with the ftate and progrefs of medicine in the North, of which we have had hitherto but little knowledge; and the well known talents of the Editors, and the interefting contents of the firft number, give us reafon to hope, that this undertaking will anfwer their views, and fatisfy the expectations of the learned. We find here, amongft the *Original Papers*, 1. *New Experiments on Refpiration and its ufe, by Prof. Abilgaard, with Remarks of Prof. Pfaff.*—Thefe experiments are directly oppofite to the generally adopted doctrine of refpiration, and Mr. Abilgaard attempts nothing lefs than to prove by them, that very little air, or none at all, enters into the lungs during infpiration; againft which Prof. Pfaff defends in his remarks the general opinion. 2. *Diffection of a drowned horfe, by J. Kuhn, with fome phyfiological remarks of Mr. Herholdt and Rafn.*—The object of this paper is chiefly concerning the air bladders that are found in large drowned animals. 3. *Experiments on Galvanifm,* are contributions to Mr. Humboldt's work on the fame fubject. 4. *Contributions to the hiftory of larvated and contagious intermittent fevers,* contain fome interefting obfervations. 5. *On Small-pox Inoculation, by Prof. Pfaff,* where he relates fome obfervations made in inoculating a great number of children in feveral villages at once. 6. *Defcription of a hooked forceps, and a perforator, with a fheath, by Dr. Scheel.* — *Short reports and extracts from letters,* include a fhort account of the mineral waters of Sweden, by Hedin, &c. *Literature of Northern Medicine and Natural Hiftory,* A review of fome differtations of Kiel and Copenhagen, and of feveral pamphlets relating a dyfentery prevailing in the neighbourhood of Kiel.

*Encyclopedie, i. e. Encyclopedia of the whole Chemiſtry, compoſed by* F. Hildebrandt, Prof. at Erlangen. Firſt Vol. Theory. Firſt Number, 1799. pp. 219. Price 14 gr. or about 2s. Erlangen, Walther.

We cannot but approve the excellent plan which the author has adopted here, in propoſing the elements of chemiſtry; and we are fully convinced that it is perfectly calculated to ſatisfy the requiſites which the vaſt progreſs of that ſcience entitles us to expect from a Philoſophical Syſtem of Chemiſtry. Mr. Hildebrandt intends to include the whole chemiſtry in a ſyſtematical, and, as far as poſſible, in a mathematical order; with ſuch a combination of theory and practice, that it may prove a very uſeful manual, not only for the philoſophic and profeſſional chemiſt, but alſo for a common reader, and for the uſe of life. With reſpect to this, the preſent work is to comprehend as well all parts of applicated as of pure chemiſtry, to the former of which a proper ſection is to be devoted. The theoretical part is to precede, to which the practical will follow, in ſuch a manner that its rules may be derived from the theory, and the reſults in the different operations explained by it. The whole is written with as much conciſeneſs as poſſible, and the paragraphs are therefore but aphoriſtic, and the quotations but few.

How far, however, the author has fulfilled this difficult and intricate taſk, which he has planned to execute, we ſhall not be able to judge till more numbers have made their appearance. In the preſent number we find, according to the plan, the theory of the different elements in general, of heat, light, oxygen, azote, atmoſpheric air, of hydrogen, and of water.

---

*J. Van Heckeren, M. D. de Osteo geneſi præternaturali, cum Tabula aenea*; 4to. pp. 125. Lugduni, Batavor. 1797. Price 2 rixd. 6 gr. or about 8s.

The Author of this learned Diſſertation treats, firſt, of the morbific oſſification which takes place in the preternatural ſwelling of the bones themſelves, and afterwards of the oſſification in the cartilage, in the membranes, and other ſoft parts of the body. From his inquiries and ſtrict obſervations of the proceſs which Nature follows in forming ſuch bony ſubſtances, it appears, Naturam in oſteogeneſi præternaturali, habita partium, quibus contingit ratione non minus ac in aliis aberrationibus *constantes* ſequi leges atque *vertum* in agendi modo ordinem ſemper ſervare.

In the accurate explanation of theſe laws the Author ſhows with much acuteneſs, how conſtant they really are, notwithſtanding the appearance of irregularity with which the morbid accumulation of bony matter ſeems to iſſue; and it appears that Nature acts here, likewiſe, according to eſtabliſhed organic regulations; and thus, what has been otherwiſe called a concrementum inorganicum, ought properly to be conſidered as a regular production of Nature. The Author then proceeds to diſcuſs, copiouſly, upon the exuberance of callus; and having examined the late opinions of the nonexiſtence of callus, he gives his own opinion, which he illuſtrates by by a very good figure of a morbific bone. His definition of the callus luxurians is the following: "Callus luxurians vocatur, qui majora copia formatus eſt, quam ad deperditam non ſolum oſſis ſubſtantiam reſtaurandum ſed etiam ad debitam ſanitati convenientem firmitatem toti oſſi tribuendam requirebatur." In accurately ſurveying a bone, where luxuriant callus has been formed, it is apparent that plaſtic Nature never performing any thing ſuperfluous, does not produce an exuberance and deformity of callus from mere luxuriance, but that it is always occaſioned by certain external and internal cauſes, by which the formative act of Nature is diſturbed and rendered irregular; how they act and influence upon the formation of the callus is amply explained by the Author.

---

*Critical Survey of the Lateſt Theories on difficult Dentition in Children.*
(Partly extracted from the Journal of Invention, Theories, &c. in German. Numb. XXXI. 1800.)

Dentition has been always conſidered as a matter of great importance in diſeaſes of children, and all the different and ſometimes dangerous affections incident to the infantile age at the time they are cutting their teeth, have hitherto been ſuppoſed by almoſt every practitioner to originate from the irritation of the gums by teeth breaking through; and the appellation of *dentitio difficilis* comprehends a ſeries of ſymptoms, that, however they may differ in their external appearance, are derived from the ſame ſource. A ſwelling and inflammation of the gums, with a great ſalivation and violent pain, and even ſpontaneous hydrophobia, a collection of pituitous matter in the breaſt, a laborious breathing, coughs, or continual vomition of bilious matter, vehement diarrhœas, convulſions, and other ſpaſmodic affections; fevers with acute exanthemata, a ſuppreſſion, or a ſuperabundant excretion of urine, or a preternatural ſharpneſs of it; an inflammation of the urethra and prepuce, with a kind of gonorrhœa; all theſe are mentioned by medical writers as immediate ſymptoms of teething, however increaſed and changed by other concomitant cauſes, as colds, ſmall pox, worms, &c.

Such was the prevailing opinion of dentition, and the ſymptoms that ſometimes attend it, till of late it has been made a matter of inquiry and diſcuſſion. Three of the moſt reſpectable authors and phyſicians of Germany, finding great difficulties in explaining thoſe ſymptoms according to the opinion which was generally adopted, made an attempt to eſtabliſh new theories on dentition, by which alſo the practice in thoſe affections is greatly influenced. Although they agree, that the mere irritation of the gingiva cannot be allowed to be the immediate cauſe of thoſe ſymptoms, yet they differ very much in the explanation and theory they reſpectively give of their nature and origin.

It is certainly undeniable that the idea of difficult dentition has been too far extended; but whether, on the other ſide, it ought to be conſidered of no immediate influence at all, is ſtill a matter of diſpute, as will appear from the obſervations annexed. It is proper now, now, however, to proceed to give a concife account of each theory; of which we fhall firft relate,

I. *Dr. Hecker's Theory,* (contained in his Magazine for Pathological Anatomy and Phyfiology. Numb. I.)

The common opinion of the act of teething in children is fubject to fome difficulties, as it cannot be well explained, how it is poffible a part, as the gums, not poffeffed of much fenfibility and irritability, only by being irritated from the teeth cutting through, fhould produce fuch violent fymptoms, as are obferved fometimes during the period of firft dentition. Another caufe ought, therefore, to be fought for, that will prove more fatisfactory in explaining, and more ufeful in directing a proper practice and cure of thofe fymptoms, to which fo many children fall a facrifice.

It is a general law founded upon facts and experience, that as foon as the organs of fecretion are difeafed or affected in a morbid manner, they fecern fuch humours as are alfo morbid and preternatural. Thus, an irritation of the liver will produce a fharp and corrupted gall; paffions often render the milk venomous; and the mildeft faliva of an irritated and angry animal becomes very malignant and poifonous. Now, in a child that is cutting teeth, many circumftances take place, which are fufficient to turn the faliva into a very powerful, malignant, and even deadly poifon. We obferve a great collection of faliva and an irritation in the mouth of a teething child; it fuffers a great deal of pain; reftlefs and fleeplefs, it is continually crying, irritated, and in a degree of paffion. This, already, may lead us to conjecture, that the faliva acquires fuch a malignity as to be able to caufe many and very dangerous fymptoms of difficult dentition.

It will, however, be ftill further proved by the following arguments: That *a corruption and acrimony of the faliva, almoft fimilar to that in the canine madnefs, is the principal caufe from which all the moft dangerous fymptoms of dentition are to be derived.*

1. Many fymptoms of dentition admit of a more natural and eafier explanation from this faliva than from the irritation only, viz. the coughs, laborious breathing, the collection of pituitous matter in the breaft, fuffocation, &c.; fwallowing it caufes vomition and diarrhœa. When it poffeffes a high degree of acrimony, or when its excretion is by any means obftructed, it produces infenfible and irritable conftitutions, hydrophobia, locked jaw, epileptic fits, &c. The acrimony being imparted to the humours, gives rife to fevers and exanthemata. The inflammatory and gonorrhoic affections of the genitals are owing to acrid faliva having thrown itfelf upon the urinary fyftem; a complication of dyfentery and dentition is confequently very dangerous, becaufe the bowels are thus likely to be doubly affected.

2. Dentition has been obferved to be flight and eafy whenever the falivation was confiderable, or falival humours evacuated by other emunctories of the body.

3. There is a great fimilarity between the fymptoms of difficult dentition dentition and thofe of real hydrophobia, apparent from the impediment in fwallowing, and other fpafmodic affections.

4. Several children, who died of difficult dentition, had bloody ftools, attended with a tenefmus, as in dyfentery. Upon diffecting the body, erofions and inflammations were found in the throat, ftomach, and inteftines, which were moft probably caufed by the acrid faliva; fomething fimilar has been noticed in the ftomach of perfons who have died of hydrophobia.

5. The cure confifts in diminifhing the irritation in the mouth, in removing the inflammation of the gums, of the falival glands, and the tonfils. To evacuate the fharp faliva, and to render it lefs noxious, are indications to which we are led by Nature itfelf. The moft difficult dentition becomes lefs dangerous when there is much falivation, vomition, and moderate diarrhœa.

The principal remedy is *the fixed cauftic alkali*, or *the volatile alkali*, which has been already recommended by Fred. Hoffmann. When this is given early enough, before the nervous fyftem is too much affected, or before too vehement fpafmodic or even apopletic fymptoms have arifen, it certainly is able to fave the life of many children. It moreover, in general, is a very ufeful remedy, whenever the organs for the fecretion of lymphatic humours being irritated and inflamed, fecern morbific fluidities; for inftance, in gonorrhœa, in fome cafes of dyfentery, catarrhous inflammations of the throat and breaft, hydrophobia, &c. Blifters applied behind the ears are of great fervice. *The incifion of the gums* can only ferve to mitigate the violent inflammation, but does by no means promote the cutting of the teeth. *Opium* fhould be avoided, or ufed at leaft with great caution, as apoplectical fits, of which many children die in dentition, are frequently brought on by it. *Extractum hyosciami* may be given inftead of it; *warm baths* have likewife great effect in removing the fpafms and pains, and in promoting falutary excretions. *Emetics* and *cathartics* are alfo fometimes very ufeful.

II. *Dr. Brandis's Theory.*

The intelligent Dr. Brandis, of Brunfwick, known by feveral excellent publications, and Tranflator of Darwin's Zoonomia, advances his opinion about the nature and origin of the dangerous fymptoms fometimes obfervable at the time of firft dentition, in his book *on Metaftases*, 1798, in German. Although he agrees with Armftrong and Hecker, that they are not to be derived only from the irritation of the nerves of the teeth, yet he rejects their theories, and rather thinks, that *a suppression of salival secretion* has the principal fhare in producing thofe fymptoms. The fecretion of faliva is much increafed by the topical irritation in the mouth, and becomes very neceffary to the conftitution of the child. When the topical irritation is too vehement, in a difficult dentition, it extends to the falival glands, and caufes a fuppreffion of the fecretion of faliva. It may be obferved, therefore, that the mouth and lips become dry and cold, in bad cafes; meanwhile, there is a great degree degree of febrile heat in other parts of the body, which is a diagnoftic fign of this dangerous difeafe.

When the fuppreffed action of the falival glands is replaced by that of the pancreas, a diarrhœa comes on, which, as it generally continues as long as the difficult dentition is accompanied by thofe fymptoms, contributes very much to diminifh the violence of them, and of the concomitant fever. But, on the contrary, when this does not take place, nervous fymptoms, convulfions, and a nervous fever arife, which having a great fimilarity with Hydrocephalus Internus, is very well defcribed by Armftrong under the name of hectic fever. That thefe nervous fymptoms cannot be afcribed to the nervous irritation arifing from the teeth cutting through, may be farther proved by the following confiderations:

1. Nervous fymptoms, arifing from a re-action of the fenforium caufed by the pain, are never removed by any material excretion, as this manifeftly happens in difficult dentition by falivation and a diarrhœa of watery humours.

2. All anodynes and antifpafmodic remedies, opiates particularly, which in convulfions proceeding from pain, prove fo very efficacious, are without any avail here, of which circumftance Dr. B. had frequent experience.

3. The incifion of the gums ought to remove the caufes of the difeafe at once, according to the theory generally believed; but in four cafes, where he faw it ufed, it was not only of no immediate utility, but in fome inftances, by additional irritation, it increafed the nervous fymptoms. A healthy child, fifteen months old, having fuffered a flight degree of cold, was fuddenly feized with all the fymptoms of difficult dentition. Emetics, mercurial purgatives, fomentations of the mouth, &c. were applied in vain. It lay fenfelefs, with its mouth open, and cold; the pulfe was hardly perceptible, and vehement convulfions came on from time to time. The gums were cut in on both fides to the teeth themfelves, after which it feemingly recovered a little; but foon after, the mouth and the lips remaining dry, pale, and the cheeks cold, the fever and convulfions returned; and though the operation was repeated, and ointments rubbed in, outfide and infide, it died the next day. The gums were found divided by the operation, and the teeth free and vifible, but no inflammation could be traced on the margin of the incifed gums. The operation fhould have certainly faved the child, if the difeafe had been dependent from the cutting of the teeth, and a falivation would have had that effect.

In examining thofe theories, the arguments fhould firft be confidered, by which they have attempted to prove that the fymptoms derived from difficult dentition are not owing to the irritation of the gums, on account of their being lefs fenfible, and not provided with nerves, as to be able to caufe thofe fymptoms; that the incifion of the gums is of no utility, and, confequently, that the nature of the fymptoms themfelves is by no means correfpondent with the quality of the caufe they are fuppofed to be occafioned by. Although the theories juft propofed are partly grounded upon thofe confiderations, confiderations, yet it may be proper to defer reviewing them till we relate Dr. Wichmann's Theory, as it is particularly urged by that gentleman.

Both Theories are certainly very ingenious and plaufible, feveral doubts, however, may be raifed againft them, to which they feem to be expofed. In the firft place, Dr. Brandis objects to Dr. Hecker's opinion, that, as the fymptoms of difficult dentition do not arife before the falivation is entirely fuppreffed, and confequently before the fecretion of any faliva is ftopped, they cannot owe their origin to its acrimony, of which quality he never could perceive any figns in a teething child. But fhould not the exiftence and action of fuch an acrid humour be proved by the erofions and inflammations that have been found in the throat, ftomach, and inteftines of children dying in a high degree of difficult dentition? However, even when this is allowed, it may be fuggefted, that it would be more proper, and agree better with our improved knowledge of animal œconomy, to derive primarily the morbific fecretion of the faliva, and the fymptoms of difficult dentition, from the fame proximate caufe which confifts in the nervous irritation, and to confider both as the congenial effect of it, and not the morbific faliva as the original caufe of thofe fymptoms. It is moreover obvious to remark, in general, that the idea of an acrimony wandering through the body, and caufing difeafes, is by no means evident, but built upon hypothetical principles.

The opinion of Dr. Brandis is founded upon his theory of metaftafes, which he has propofed in the work above mentioned. According to this, the idea of a metaftafis ought to be thus conceived: "When certain actions in any organ, or fyftem of organs, ceafe, or are by any means diminifhed, they muft be replaced by another action in another organ or fyftem of organs of the body, dependent from the former action. The firft may be called the *original*; the fecond, the *vicegerent action*." In dentition, now, the fecretion of faliva is the original action of the falival fyftem, caufed by the topical irritation in the mouth, which in a teething child is become neceffary to its conftitution. If that action happens to be ftopped, a vicegerent action is excited in another organ; and if this takes place in the nervous fyftem, all thofe dangerous fymptoms appear which are to be attributed to difficult dentition. It is, however, not requifite, and would lead us too far, to enter into the particulars of this ingenious theory; it may fuffice to obferve, that the fuppreffion of falival fecretion might be more naturally confidered as the effect of nervous irritation, which by its violence, at the fame time produces the other fymptoms of difficult dentition. This explanation is fuitable to a known law of the animal œconomy, according to which, fecretions are increafed by a moderate degree of irritation, and fuppreffed whenever it becomes too vehement. When falivation, therefore, is not fuppreffed, but rather increafed, and the fymptoms without danger, the irritation of the nervous fyftem is moderate, which being relatively vehement, muft be looked upon as the common caufe of both,

 the the suppression of saliva as well as of the violence of the symptoms. Salivation, however, may be of some service in dentition, by rendering the gums tender, and more easily to be cut through.

Anodynes and narcotics, whose efficacy is denied here by Dr. B. have generally been found to be very useful by other eminent practitioners; and even Dr. Hecker recommends the Extractum Hyoscíami, though he ventures not to give opiates, which, however, in some instances, may prove likewise very useful.

[ To be continued. ]

---

*Almanach oder Taschenbuch*; i. e. *Almanack or Pocket-Book, for Chemists and Apothecaries, for the Year* 1800. Weimar. 8vo. Price 16 gr. or about 2 s. 6 d.

THE Editor of this useful publication, which for many years has maintained its credit with the public, is Professor Göttling, of Jena, whose great merits in chemistry are well known, and justly respected. It consists of a common Almanack, where, instead of the saints, the names of celebrated chemists are inserted; to which is added an account of those pharmaceutical operations that are to be performed in each month. The present volume gives a continuation of the revisal of operations and experiments begun in the Almanack of last year, for the purpose of accommodating the foregoing volumes to the present state of chemistry; some new experiments of the author are likewise subjoined. The present Almanack reviews those of 1782—85. The whole is concluded with accurate reviews of new chemical and pharmaceutical publications.

MEDICAL

